# Patterns of Skin Picking in Skin Picking Disorder: Ecological Momentary Assessment Study

**DOI:** 10.2196/53831

**Published:** 2024-07-18

**Authors:** Christina Gallinat, Markus Moessner, Maximilian Wilhelm, Nancy Keuthen, Stephanie Bauer

**Affiliations:** 1 Center for Psychotherapy Research University Hospital Heidelberg Heidelberg Germany; 2 German Center for Mental Health (DZPG), partner site Mannheim-Heidelberg-Ulm Heidelberg Germany; 3 Trichotillomania and Excoriation Disorder Program Center for OCD and Related Disorders Massachusetts General Hospital Boston, MA United States; 4 Heidelberg University Heidelberg Germany

**Keywords:** skin picking disorder, ecological momentary assessment, EMA, body-focused repetitive behavior, obsessive-compulsive spectrum, skin, dermatology, mental health, assessment, mobile phone

## Abstract

**Background:**

Skin picking disorder (SPD) is an understudied mental illness that is classified as a body-focused repetitive behavior disorder. Literature suggests that pathological skin picking is strongly integrated into the daily lives of affected individuals and may involve a high degree of variability in terms of episode characteristics, frequency, and intensity. However, existing data on the phenomenology of SPD are limited and typically involve retrospective assessments, which may fail to accurately capture the behavior’s variability.

**Objective:**

This study aimed to investigate skin picking in the daily lives of individuals with SPD by using ecological momentary assessment (EMA). The first aim focused on the description of skin picking patterns (eg, characteristics, intensity, and distribution of episodes and urges), and the second aim explored differences in characteristics and patterns between automatic and focused skin picking.

**Methods:**

Participants were recruited online and underwent a web-based screening, a diagnostic telephone interview, and a comprehensive online self-report questionnaire before participating in an EMA protocol. The latter included 10 consecutive days with 7 pseudorandom, time-contingent assessments per day between 8 AM and 10 PM. The EMA questionnaire assessed the current skin picking urge, the occurrence of the behavior, and a detailed assessment of the episodes’ characteristics (eg, length, intensity, and consciousness) if applicable.

**Results:**

The final sample consisted of 57 participants, who completed at least 70% of the scheduled assessments (n=54, 94.7% female: mean age 29.3, SD 6.77 years). They completed 3758 EMAs and reported 1467 skin picking episodes. Skin picking occurred frequently (mean 2.57, SD 1.12 episodes per day and person) in relatively short episodes (10-30 min; 10 min: n_episodes_=642, 43.8%; 20 min: n_episodes_=312, 21.3%; 30 min: n_episodes_=217, 14.8%), and it was distributed quite evenly throughout the day and across different days of the week. Focused and automatic episodes were relatively balanced across all reported episodes (focused: n_episodes_=806, 54.9%) and over the course of the day. The analyses showed statistically significant differences between self-reported triggers for the different styles. Visual or tactile cues and the desire to pick the skin were more important for the focused style (visual or tactile cues: mean focused style [*M*_f_]=4.01, SD 0.69 vs mean automatic style [*M*_a_]=3.47, SD 0.99; *P*<.001; SMD=0.64; desire to pick: *M*_f_=2.61, SD 1.06 vs *M*_a_=1.94, SD 1.03; *P*<.001; SMD=0.82), while boredom and concentration problems were more prominent in automatic skin picking (boredom: *M*_f_=1.69, SD 0.89 vs *M*_a_=1.84, SD 0.89; *P*=.03; SMD=–0.31; concentration problems: *M*_f_=2.06, SD 0.87 vs *M*_a_=2.31, SD 1.06; *P*=.006; SMD=–0.41).

**Conclusions:**

These results contribute to an enhanced understanding of the phenomenology of SPD using a more rigorous assessment methodology. Our findings underscore that picking can impact affected persons multiple times throughout their daily lives.

**Trial Registration:**

German Clinical Trials Register DRKS00025168; https://tinyurl.com/mr35pdwh

## Introduction

Skin picking disorder (SPD) is a mental disorder, which is characterized by the body-focused repetitive behavior (BFRB) of manipulating one’s own skin including, for example, squeezing, scratching, or rubbing—summarized as “skin picking” [1]. With a lifetime prevalence of 1.4% to 3.1% [1,2], SPD is not a rare disorder, even though it received comparatively little attention in research and clinical practice so far.

Until now, there has been little research on the phenomenology of SPD, and the existing research is of questionable validity as it often entails retrospective reporting, so the clinical picture has not been described in sufficient detail to date. This hinders a well-grounded understanding of the disorder as well as the development of specific treatment options.

Few previous studies have described skin picking in terms of the frequency and episode length; for example, one study reported a median of 38 minutes for skin picking per day (range 1-360 min), while another found a mean of 8 (SD 22) episodes per day with an average length of 21 (SD 42) minutes [3,4]. In a more recent study, 78% of participants reported that they typically have 1 to 5 episodes per day and that most episodes are shorter than 30 minutes. Moreover, the majority reported that they picked their skin almost every day [5]. Meanwhile, data on high-risk times throughout the day are very scarce, with only 1 small study reporting such data (n=31) [6]. However, the small number of studies and the large variability among the results suggest a need for additional and more rigorous investigations.

In addition to episode characteristics, different styles of skin picking characterized by the extent of awareness during behavior were examined. “Focused skin picking” is hypothesized to occur more intentionally and in response to urges or difficult emotions, whereas “automatic skin picking” takes place without awareness and is supposed to be associated with certain (routine) situations and passive activities [7]. So far, little is known about the distribution of automatic and focused skin picking within and between individuals, other than that there seems to be high variability. However, a recent study reported a shift from focused skin picking toward more automatic skin picking with increasing age [8].

In terms of episode triggers, previous studies identified certain internal and external states commonly precipitating skin picking behavior. Commonly reported triggers are affective states (eg, tension or boredom), visual and tactile perceptions of skin irregularities, passive activities, and certain situations or places (eg, waiting, reading, or bathroom) [9-12]. Unfortunately, there is currently almost no data available on the distribution of skin picking and skin picking urges over the course of a day and a week.

Moreover, the existing studies on skin picking phenomenology include crucial shortcomings due to their cross-sectional and retrospective designs. It is well known that retrospective assessments imply a high risk of systematic biases, caused by the way memories are stored and retrieved [13]. Moreover, these designs are not able to capture dynamic processes and to identify specific variations, for example, in behavioral patterns throughout the day or week. Both of these issues are relevant to studies on SPD phenomenology. For example, the large range in the number and length of skin picking episodes in former studies indicates that it is critical to examine the distribution and characteristics of the behavior and to explore the role of intraindividual and interindividual variability in the behavior. In the clinical setting, affected individuals often report that the behavior can strongly vary from day to day—depending on a multitude of factors, for example, such as being in company versus alone or at work versus at home. These differences are masked in retrospective studies when the average time spent on skin picking in the last 2 weeks is assessed.

In addition, retrospective studies usually do not allow a reliable assessment and differentiation of characteristics of different styles of skin picking, which are characterized by the extent of awareness during skin picking. Moreover, the distribution of focused versus automatic skin picking as well as the link between specific triggers and different skin picking styles have not been investigated in detail. Of note, as most individuals with skin picking show a mixture of both styles, the retrospective assessment of separate triggers for automatic versus focused episodes would be very likely biased. However, the detailed investigation of skin picking styles and the associated triggers can serve as a solid basis for the specific selection and adaptation of interventions and behavioral strategies for certain risk situations or skin picking styles.

A promising method to comprehensively investigate processes of skin picking behavior is ecological momentary assessment (EMA) [14]. Momentary assessments within the daily life of individuals provide the opportunity to study dynamic processes in real time while minimizing retrospective biases. Since EMA allows a more detailed assessment of behavioral processes and implies a high ecological validity, the method received much attention in psychological research in the last 2 decades and was successfully applied by numerous studies in the investigation of different psychopathologies (eg, anxiety, substance use, or eating disorders) [15-17].

For skin picking research, EMA is a promising tool for reliably investigating the distribution as well as characteristics of skin picking episodes. The analysis of these data then affords an understanding of the course of skin picking behavior throughout the day and week in detail and identifies high-risk times and related circumstances. To the best of our knowledge, EMA has not yet been applied to investigate these research questions in SPD. Therefore, the main objective of this study was to investigate skin picking in the natural environment of individuals having SPD using EMA. Such data are urgently needed for a more comprehensive description and understanding of the phenomenology and mechanisms of this comparatively new disorder.

The study followed 2 aims: the first aim was to describe skin picking patterns in the daily lives of the participants (eg, number, length, intensity, distribution of skin picking episodes, distribution of skin picking urges, or self-reported triggers).

The second aim of this study was to explore differences between automatic and focused skin picking concerning distributions (eg, daytime), characteristics of the episodes (eg, length or intensity), and self-reported triggers.

## Methods

### Procedures

Participants were recruited between November 2021 and May 2022 through support groups and online via mailing lists, specific forums, and social media. Inclusion required a minimum age of 18 years; satisfaction of the *Diagnostic and Statistical Manual of Mental Disorders, Fifth Edition* (*DSM-5*) criteria for SPD; and provision of informed consent for study participation. The inclusion of participants involved three stages of assessment: (1) an initial web-based screening, which assessed sociodemographic information and skin picking symptoms; (2) a diagnostic interview via telephone, in which the *DSM-5* criteria for SPD were assessed; and (3) a web-based self-report questionnaire (baseline) for those assessed to be eligible in the interview.

EMA sampling started on the day after completion of the baseline questionnaire. The assessment period comprised 10 consecutive days with 7 pseudorandom, time-contingent assessments per day between 8 AM and 10 PM. In addition, participants were asked to record additional skin picking episodes (event contingent recording). The prompts were sent to the participants’ smartphones via text message, which contained a link to the EMA questionnaire. Additional records could be made via the web-based study platform. The time and event contingent EMA records took at most 5 minutes. All assessment procedures were conducted with the software ASMO [18].

### Measures

#### Screening

The screening questionnaire included sociodemographic variables and the German version of the Skin Picking Scale-Revised (SPS-R) [19,20]. The scale assesses skin picking severity over the past week and consists of 8 items that can be split into 2 subscales: symptom severity and impairment. A global score can also be calculated. All items are rated on a 5-point Likert Scale from 0 (eg, “none”) to 4 (eg, “extreme”). The internal consistency of the total scale was high in this study (α=0.84; subscales: symptom severity: α=0.77 and impairment: α=0.85).

#### Diagnostic Interview

To assess the *DSM-5* criteria for SPD, semistructured interviews based on a BFRB module (personal communication with L Mehrmann, February 2021) for the DIPS Open Access Diagnostic Interview for Mental Disorders were conducted via telephone [21]. The interviews were carried out by the first author (CG) and a student worker, who was trained and continuously supervised.

#### Baseline Measures

##### Overview

The baseline questionnaire contained the following assessment instruments.

##### Skin Picking Severity

The current skin picking severity was assessed in the baseline questionnaire with the SPS-R described above [19,20].

##### Impairment due to Skin Picking

Skin picking–related impairment was assessed with the German translation of the Skin Picking Impact Scale (SPIS) [22,23], which refers to the last week and contains 10 items capturing potential impairments due to skin picking (eg, feeling unattractive, ashamed, or not being able to do certain things due to skin picking) rated on a 5-point Likert scale (0: “not at all”; 4: “severe”). The internal consistency of the SPIS was excellent in this study (α=0.90).

##### Modes of Skin Picking

Different styles of skin picking (focused vs automatic) were assessed with the German version Milwaukee Inventory for the Dimensions of Adult Skin Picking (MIDAS) [7]. We translated the scale in a former study following generally accepted recommendations including backtranslation and approval by one of the authors of the original scale (DW Woods) [24]. The scale consists of 12 items, which are rated from 1 “not true for any of my skin picking” to 5 “true for all of my skin picking”. Both subscales (focused, automatic) contain 6 items and showed an acceptable internal consistency of α=0.62.

##### Depressive Symptoms

Depressive symptoms were captured using the Patient Health Questionnaire-9 (PHQ-9) [25]. The scale contains 9 items, which are rated on a Likert scale from 0 (“not at all”) to 3 (“almost every day”) in reference to the last 2 weeks. The scale showed a good internal consistency in our study (α=0.84).

##### Anxiety

Symptoms of generalized anxiety disorders were assessed with the Generalized Anxiety Disorders-7 (GAD-7) [26]. The Cronbach α was 0.84.

#### EMA Questionnaire

The EMA assessments included urge intensity (1: “no urge” to 5: “very strong”) and skin picking occurrence since the last assessment (yes or no). If skin picking occurred, additional questions assessed the following: intensity of skin picking (1: “very weak” to 5: “very strong”), length of the episode (12 options: about 10, 20, 30, …, 120 min), awareness at episode onset (“Did you notice when you started picking your skin?”; yes or no), and perceived triggers (“What contributed to your skin picking?”). For the last question, seven items had to be rated on a 5-point Likert scale: (1) visual or tactile cues, (2) itching, (3) tension, (4) boredom, (5) difficulties concentrating on a task, (6) desire for skin picking, (7) certain routine (eg, evening routine), and (8) other (text field).

### Statistical Analyses

Patterns of skin picking were analyzed using descriptive statistics. Frequencies for the number of episodes with certain characteristics (length, time of occurrence, or consciousness) were analyzed across all individuals and episodes. To control for the unequal number of skin picking episodes reported per person, mean scores within each person were calculated for urge intensity, episode intensity, and the rating of specific triggers. The average scores of the person means are reported. The distribution of skin picking urges as well as the distribution and characteristics of automatic and focused skin picking were also analyzed descriptively. *t* tests (2-tailed) for paired samples were calculated to test differences between focused and automatic episodes. Focused and automatic episodes were classified based on the yes or no question “Did you notice when you started picking your skin?” Differences were quantified using SMD. Analyses were performed in R (version 4.1.2; R Development Core Team, 2021) and with SPSS Statistics (version 29.0; IBM).

### Ethical Considerations

All study procedures adhered to the latest version of the Declaration of Helsinki and were approved by the ethics committee of the Medical Faculty of Heidelberg University (S-222/2021). The trial was registered at the German Clinical Trials Register before recruitment started (DRKS00025168).

Participants provided informed consent for this study ahead of the initial screening and were able to discontinue participation at any time. Data are pseudonymized and can be subsequently matched to the respective persons only by authorized personnel. The data are anonymized as soon as possible after completion of the analyses.

All participants were provided with a €15 (approximately US $16) compensation in the form of a gift voucher for a bookstore. Additionally, if participants achieved an EMA completion rate of at least 70% (49 assessments), the voucher was upgraded to €50 (approximately US $54).

## Results

### Sample Description

**Figure 1:**
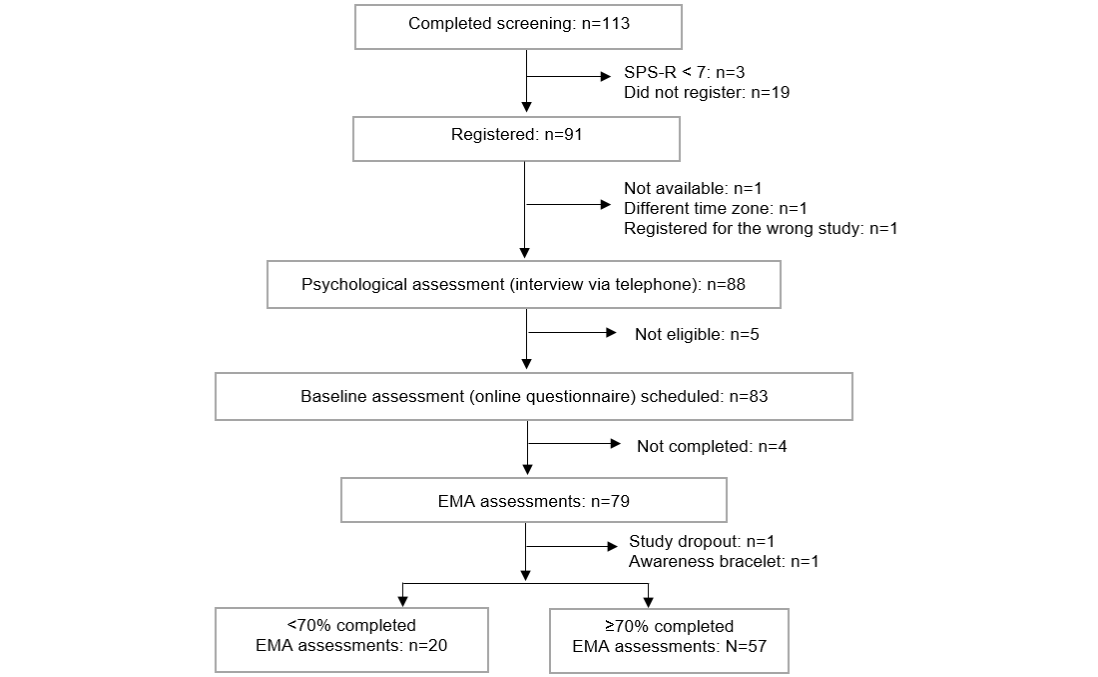
Participant flow. EMA: ecological momentary assessment; SPS-R: Skin Picking Scale-Revised.

Overall, 113 individuals completed the screening questionnaire. Of these, 79 (69.9%) participants started the EMA assessments. Further, 1 person dropped out during the EMA period and 1 person was excluded from the final analysis due to wearing an awareness bracelet, which vibrates when touching certain body areas for the prevention of skin picking. Overall, 57 (74%) out of 77 answered at least 70% of all scheduled EMA questionnaires (ie, at least 49 assessments). The participant flow is shown in Figure 1.

Participants of the final sample (n=57) completed 65.93 (SD 7.24) EMAs on average, with a range of 51 to 99 per person. Frequencies above the number of scheduled time-contingent assessments (n=70) result from additional entries made by participants on their own initiative (event-contingent records).

### Participants

The majority of participants were female (54/57, 94.7%) with a mean age of 29.3 (SD 6.77) years. About half (28/57, 49.1%) of the participants were employed and one-third (18/57, 31.6%) were university students. The sample showed a PHQ-9 mean score of 11.63 (SD 5.41), indicating moderate depressive symptoms; a GAD-7 mean score of 9.63 (SD 4.85), indicating mild to moderate anxiety; and a mean SPS-R score of 18.00 (SD 4.00), indicating substantial SPD severity. The participants in the analyzed sample do not differ from the participants who were excluded from the analyses due to the low EMA completion rate (less than 49 assessments, <70%). *t* tests (2-tailed) for independent samples and *χ*-quadrat tests did not yield any statistically significant differences in terms of the assessed sociodemographic and clinical variables (all *P*>.05). A detailed overview of the sample characteristics is given in Table 1.

**Table 1:** Sample characteristics.

Characteristics		Total EMA^a^ sample (n=77)		EMA≥70% (n=57)		EMA<70% (n=20)
Female sex, n (%)		74 (96.1)		54 (94.7)		20 (100)
Age (years), mean (SD)		28.84 (6.51)		29.3 (6.77)		27.3 (5.74)
**Education, n (%)**						
	Still in school	1 (1.3)	—^b^		1 (5)	
	Middle secondary	8 (10.4)	6 (10.5)		2 (10)	
	Highest secondary	27 (35.1)	19 (33.3)		8 (40)	
	University	41 (53.2)	32 (56.1)		9 (45)	
**Occupational status, n (%)**						
	Employed	40 (51.9)	28 (49.1)		12 (60)	
	Trainee	1 (1.3)	1 (1.8)		—	
	School student	1 (1.3)	—		1 (5)	
	University student	23 (29.9)	18 (31.6)		5 (25)	
	Housewife or househusband	3 (3.9)	3 (5.3)		—	
	Retired	2 (2.6)	1 (1.8)		1 (5)	
	Unemployed	2 (2.6)	1 (1.8)		1 (5)	
	Other	5 (6.5)	5 (8.8)		—	
**Family status**						
	Single, n (%)	34 (44.2)	22 (38.6)		12 (60)	
	In a relationship, n (%)	25 (32.5)	20 (35.1)		5 (25)	
	Married, n (%)	15 (19.5)	12 (21.1)		3 (15)	
	Separated or divorced, n (%)	2 (2.6)	2 (3.5)		—	
	Other, n (%)	1 (1.3)	1 (1.8)		—	
	PHQ-9^c^, mean (SD)	11.95 (5.6)	11.63 (5.41)		12.15 (5.86)	
	GAD-7^d^, mean (SD)	10.04 (4.64)	9.63 (4.85)		10.5 (4.01)	
	SPS-R^e^, mean (SD)	17.69 (3.98)	18 (4)		16.7 (3.87)	
	SPIS^f^, mean (SD)	23.45 (8.57)	23.33 (8.94)		23.8 (7.61)	
	MIDAS focused^g^, mean (SD)	19.9 (3.92)	19.7 (3.9)		20.45 (4.05)	
	MIDAS automatic^h^, mean (SD)	18.18 (3.63)	18.49 (3.58)		17.3 (3.74)	

^a^EMA: ecological momentary assessment.

^b^Not available.

^c^PHQ-9: Patient Health Questionnaire-9, depressive symptoms.

^d^GAD-7: generalized anxiety disorders-7.

^e^SPS-R: Skin Picking Scale-Revised.

^f^SPIS: Skin Picking Impact Scale.

^g^MIDAS focused: Milwaukee Inventory for the Dimensions of Adult Skin Picking, focused skin picking.

^h^MIDAS automatic: Milwaukee Inventory for the Dimensions of Adult Skin Picking, automatic or unconscious skin picking.

### Number and Distribution of Episodes

In total, 57 participants completed 3758 EMAs and reported 1467 skin picking episodes during the EMA period of 10 days. Altogether, 1351 (92.1%) episodes were reported in time-based assessments and only 116 (7.9%) in event-based assessments. On average, participants reported a mean number of 2.57 (SD 1.12; range 0.8-5.4) episodes per day.

Slightly more than half of the sample (32/57, 56.1%) reported episodes on each day of the 10‑day EMA phase, while 28.1% (n=16) reported 1 day without skin picking and 15.8% (n=9) had 2, 3, or 4 days without skin picking.

**Figure 2:**
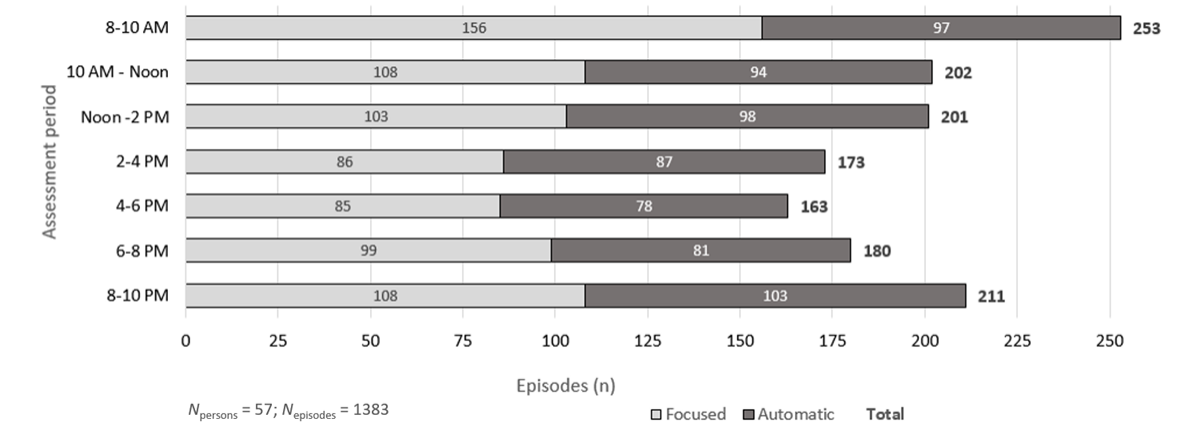
Episode distribution throughout the day (event- and time-based EMAs; 8 AM-10 PM). EMA: ecological momentary assessment.

Skin picking episodes were relatively evenly distributed throughout the day. Small peaks in the number of episodes emerged in the first (8-10 AM; n_episodes_=253, 17.3% of all 1467 episodes) and the last (8-10 PM; n_episodes_=211, 14.4% of all 1467 episodes) regular assessment period of each day. The number of episodes over the course of a day is shown in detail in Figure 2 and Table 2. It should be noted that to avoid a biased comparison between time-based and event-based surveys, only the periods covered by the time-based assessment are presented.

**Table 2:** Urge and episode parameters over the day^a^.

Time	All episodes (N=1467), n (%)	Focused episodes (n=806), n (%)	Automatic episodes (n=661), n (%)	Intensity of episodes (n=1467)*,* mean (SD)	Urge intensity EMA^b^ with episodes (n=1467), mean (SD)	Urge intensity EMA without episodes (n=2291), mean (SD)
8-10 AM	253 (17.2)	156 (19.35)	97 (14.67)	2.61 (0.87)	2.53 (0.98)	1.78 (0.79)
10 AM to Noon	202 (13.8)	108 (13.4)	94 (14.22)	2.38 (0.72)	2.54 (0.92)	1.92 (0.73)
Noon to 2 PM	201 (13.7)	103 (12.78)	98 (14.83)	2.19 (0.78)	2.75 (0.86)	1.87 (0.68)
2-4 PM	173 (11.8)	86 (10.67)	87 (13.16)	2.43 (0.86)	2.89 (0.84)	1.83 (0.71)
4-6 PM	163 (11.1)	85 (10.55)	78 (11.8)	2.43 (0.88)	2.90 (0.95)	1.92 (0.76)
6-8 PM	180 (12.3)	99 (12.28)	81 (12.25)	2.43 (0.78)	3 (0.85)	1.92 (0.71)
8-10 PM	211 (14.4)	108 (13.4)	103 (15.58)	2.77 (0.74)	3.02 (0.88)	1.94 (0.76)

^a^Only periods of time-based assessments are listed. The total N refers to all registered episodes (N_episodes_=1467, N_persons_=57). Intensity scores are average scores of person-wise means.

^b^EMA: ecological momentary assessment.

### Weekdays

The episodes were quite evenly distributed over the days of the week. Across all participants, the average number of episodes per day ranged between 2.20 for Saturdays and 2.77 for Mondays and Tuesdays (Monday: mean 2.77, SD 1.78; Tuesday: mean 2.77, SD 1.65; Wednesday: mean 2.66, SD 1.50; Thursday: mean 2.54, SD 1.50; Friday: mean 2.53, SD 1.59; Saturday: mean 2.20, SD 1.50; and Sunday: mean 2.57, SD 1.66).

### Length and Intensity

Of all 1467 episodes, participants indicated the shortest selectable length (approximately 10 minutes) in 43.8% (n=642), 20 minutes in 21.3% (n=312), and 30 minutes in 14.8% (n=217; Table 3). Only 9 (15.8%) participants reported any episode of 60 minutes or longer and only 6 (10.5%) reported episodes of at least 90 minutes.

Table 2 displays the distribution of focused and automatic episodes as well as episode intensity and urge intensity over the course of the day.

The reported intensity of the episodes across all subjects was on average 2.55 (SD 1.11; 2: “mild”, 3: “medium”). Throughout the day, the intensity of the episodes was quite stable. The average person means in the regular EMA phase (8 AM-10 PM) varied between 2.19 (SD 0.78; noon to 2 PM) and 2.77 (SD 0.74; 8-10 PM). Slightly higher average intensities were reported in the evening and the morning (see Table 2).

Overall, in terms of intensity, most episodes were rated as very mild (288/1467, 19.6%), mild (n=448, 30.5%), or medium (n=426, 29%). Participants rated 16.4% (n=240) of the episodes as severe and 4.4% (n=65) as very severe. Further, 10 (17.5%) participants did neither report severe nor very severe episodes.

**Table 3:** Length of episodes^a^.

Approximate length	Episodes), n (%)
10 min	642 (43.8)
20 min	312 (21.3)
30 min	217 (14.8)
40 min	97 (6.6)
50 min	39 (2.7)
60 min	64 (4.4)
70 min	14 (0.9)
80 min	26 (1.8)
90 min	22 (1.4)
100 min	6 (0.4)
110 min	2 (0.1)
120 min	26 (1.8)

^a^All episodes reported in time- and event-based ecological momentary assessments (N_episodes_=1467; N_persons_=57).

### Urge Intensity

The mean urge intensity (average scores of person-wise means) in assessments with reported episodes varied between 2.53 (SD 0.98) in the morning (8-10 AM) and increased in small increments throughout the day with the highest mean being 3.02 (SD 0.88) in the evening (8-10 PM). So, the average urge intensity varied between mild (“2”) and medium (“3”) and was significantly higher in assessments with reported episodes (mean 2.84, SD 0.71) compared to those without episodes (mean 1.89, SD 0.65; *t*_56_=12.31; *P*<.001; SMD=1.63). The average scores for the urge intensity per period are shown in Table 2.

### Episode Characteristics

#### Consciousness

Participants reported a conscious onset of the behavior in 54.9% (n=806; “focused episodes”) and an unconscious onset in 45.1% (n=661; “automatic episodes”) of all 1467 episodes. Most participants reported both types of episodes (49/57, 86%). One-third of participants (n=19) reported 75% or more focused episodes and 8 (14%) patients of these reported exclusively focused episodes. A proportion of 75% or more automatic episodes was indicated by 8 (14%) participants, and overall, it ranged between 0% and 98.2% (median 39.3, IQR 14.2-63.3).

Across all participants, the ratio between these 2 modes was relatively balanced throughout the day, with focused episodes occurring slightly more often. However, comparatively more focused episodes occurred in the morning (8-10 AM). Details are shown in Table 2.

Focused and automatic episodes did not differ significantly in terms of the intensity of the behavior or urge intensity (intensity: mean focused style [*M*_f_]=2.56, SD 0.62; mean automatic style [*M*_a_]=2.45, SD 0.78; *t*_48_=1.52, *P*=.14; urge intensity: *M*_f_=2.86, SD 0.77; *M*_a_=2.90, SD 0.86; *t*_48_=–0.19, *P*=.85).

#### Self-Reported Triggers

Across all participants, the highest average values resulted for visual or tactile cues (eg, felt or seen something on the skin; mean 3.64, SD 1.26), tension (mean 2.63, SD 1.29), and habit (mean 2.71, SD 1.45).

Comparisons between focused and automatic episodes showed higher scores in focused episodes for visual or tactile cues as well as for the item “wanted to pick the skin” (SMD=0.64 and 0.82, respectively)*.* In contrast, boredom and problems with concentration achieved higher scores in automatic episodes (SMD=–0.31 and –0.41, respectively)*.*

In the “other” category, additional conditions were mentioned in 97 episodes: working or being at the PC, talking on the phone, smartphone time, reading, watching television, driving, showering, encountering a mirror, physical fatigue or tiredness, hunger, emotional discomfort, and social situations or conflicts. Scores are displayed in Table 4.

**Table 4:** Self-reported triggers^a^.

Trigger	Total (N=57), mean (SD)	Focused (n=49), mean (SD)	Automatic (n=49), mean (SD)	*t* test^b^ (*df*)	*P* value	SMD
Visual or tactile cues	3.64 (1.26)	4.01 (0.69)	3.47 (0.99)	4.482 (48)	<.001	0.64
Tension	2.63 (1.29)	2.67 (0.89)	2.84 (0.97)	–1.532 (48)	.13	–0.22
Boredom	1.69 (1.03)	1.69 (0.89)	1.84 (0.89)	–2.187 (48)	.03	–0.31
Problems with concentration	2.17 (1.31)	2.06 (0.87)	2.31(1.06)	–2.847 (48)	.006	–0.41
Wanted to pick the skin	2.14 (1.27)	2.61 (1.06)	1.94 (1.03)	5.753 (48)	<.001	0.82
Habit or routine	2.71 (1.45)	2.82 (1.07)	2.56 (1.10)	1.818 (48)	.08	0.26
Itch	1.67 (1.09)	1.75 (0.94)	1.81 (1)	–0.594 (39)	.556	–0.09

^a^Answers rated on a 5-point Likert scale (1: not at all; 5: extremely). *t* test results refer to comparisons of the average scores of person-means in focused and automatic episodes (n_episodes_=1295, n_persons_=49). Further, 8 persons were excluded from the comparison as they reported no automatic episodes. “Habit/routine” relates to the item “I picked my skin out of a routine (eg, after arriving home or during the evening bath routine).”

^b^2-tailed.

## Discussion

### Principal Findings

SPD has now been officially recognized as a separate disorder for more than 10 years. However, despite increased research efforts, there is still a lack of studies on the phenomenology of the disorder. To our knowledge, this is the first study to investigate skin picking behavior by using EMA in the daily life of people with SPD.

The results document in several ways how strongly the behavior is interwoven with the everyday life of affected individuals. For example, 56.1% (32/57) reported that they experienced no day without skin picking within the 10-day study phase, but only 15.8% (n=9) reported 2 to 4 days without skin picking. In other words, skin picking occurred almost every day. In addition, participants reported an average of 2.6 episodes per day (range 0.8-5.4), suggesting that the behavior is not limited to 1 daily episode, but occurs several times a day and continuously influences daily life. These results are consistent with the results of 2 retrospective studies reporting also several episodes per day [3,5]. The continuity of the behavior is also reflected by the results over the course of the day and the week. Throughout the day, episodes were more or less evenly distributed, with only small peaks in the morning and evening. Similarly, the average urge intensity varied only slightly over the monitored periods and ranged constantly between weak and medium, with values in the evening being somewhat higher. However, as expected, the urge intensity was considerably higher in assessments with reported episodes compared to those without. Regarding the frequency of the episodes, there were also only a few small differences between the different weekdays. The lowest average number of episodes was reported for Saturdays and the highest for Mondays and Tuesdays, but the differences between other weekdays were quite small. Overall, data regarding the skin picking urges and behavior indicate that both are experienced frequently by affected individuals.

In terms of the episode characteristics, it is important to note that 43.8% (n_episodes_=642/1467) were no more than 10 minutes long and 80% (n_episodes_=1171) of the episodes were no longer than 30 minutes, so the results suggest rather short, but frequent episodes. This is also in line with previous studies reporting that the majority of episodes are under 30 minutes [3,5]. However, short episodes are not necessarily mild since the skin can be severely damaged in just a few minutes.

Regarding consciousness of the episodes, the results show groups of individuals with a quite high preponderance (eg, ≥75% of episodes) of a focused (19/57, 33%) or automatic (8/57, 14%) style. A unilateral skin picking style, where individuals predominantly (>95% of all episodes) show either automatic or focused skin picking, was relatively rare (automatic: 2/57, 4%; focused: 8/57, 14% of the sample).

However, the ratio between focused and automatic episodes was relatively balanced, although there were clear differences between individuals. Overall, more participants showed a tendency toward a focused style. The minor predominance of focused skin picking is also consistent with the results of a recent study that similarly found a slight dominance of focused skin picking for middle adulthood [8].

In recent years, different studies tried to identify different skin picking subtypes between individuals regarding various characteristics (eg, symptom presentation and styles of skin picking, or neurobiology), but nevertheless, this research is still in its beginning [7,27-30]. However, as research shows that most people with SPD show both styles of skin picking, there is an obvious necessity to understand the different types of pathological skin picking to develop prevention and intervention strategies specifically for automatic and focused skin picking. This is especially the case because the onset and course of an automatic episode can strongly differ from focused episodes necessitating different coping strategies matched to the specific picking style.

The results showed statistically significant differences between self-reported triggers for automatic and focused episodes: visual or tactile cues and the desire to pick the skin (item “wanted to pick”) played a more important role in focused episodes, while boredom and problems with concentration were more related to automatic episodes. Other triggers (eg, tension or itch) did not differ between the 2 modes of skin picking. The largest difference was found for the trigger desire (“wanted to pick”; SMD=0.82). Of note, the results do not provide any evidence that 1 of the 2 styles is associated more strongly with tension than the other.

### Strengths and Limitations

Overall, the results offer useful insights into the nature, frequency, distribution, and intensity as well as specific triggers of skin picking. They also provide important starting points for future studies that should investigate these aspects in more detail. However, our results should be interpreted in light of the specific strengths and limitations of this study. The latter may include a bias due to the self-selection of the participants. It is likely a rather specific sample of individuals, who are willing to track their skin picking for a period of 10 days several times a day. However, our data suggest a substantial impairment in terms of skin picking severity, depression, anxiety, and skin picking–related impairment.

Another limitation results from the assessment method since self-observation and tracking skin picking can also increase the awareness and therefore the controllability of the behavior. Moreover, is it also discussed that the registration of an episode may serve a punishing function due to the extra effort to record it so that the likelihood of the behavior is reduced. These mechanisms could have produced 2 biases in this study: first, the number of automatic episodes could be underestimated due to the increased awareness during this study. Second, the frequency and intensity of the behavior may have been reduced by the continuous monitoring within this study’s period.

Also, the assessment started regularly with the question “Have you picked your skin since the last assessment?” This could have caused a bias toward more reported episodes in the first period of the day as individuals might also report skin picking, which occurred in the night before. Consequently, the total number of the period between 8 and 10 AM should be interpreted cautiously.

Another limitation refers to the assessment of the episode length, which was assessed by multiple choice with options in steps of 10 minutes. The shortest selectable option was “about 10 minutes,” but during this study, we received feedback from participants that their episodes were much shorter. However, this also leads us to the open question of what constitutes a skin picking episode and if micro episodes might play an important role. In addition, we know from clinical work that some people report that the behavior occurs almost constantly throughout the day. In this context, the question arises, whether and for whom it makes sense to divide the behavior into episodes. In this study, participants were forced to report behavioral episodes, but it remains unclear what participants have defined as an episode for themselves and if they tracked microepisodes. Future research needs to address these issues by applying an even tighter, more precise measurement of behavior, but this will also need to take the abovementioned difficulty of measurement reactivity into account.

Despite these limitations and the need for further research, this study also has some important strengths. To the best of our knowledge, this is the first study using EMA to assess skin picking, and it is also the first EMA study in the field of pathological BFRBs in general. It provides new insights into the phenomenology of the SPD allowing for a more reliable and accurate description of skin picking in the everyday life of affected individuals, which is essential for a comprehensive understanding of SPD of this relatively newly defined disorder. The study clearly demonstrates the advantages of measurement via EMA, since behavioral parameters could be assessed that cannot be measured at all—or only with considerable distortions—in retrospective assessments. Furthermore, this study was conducted with a sample of individuals fulfilling the diagnostic criteria for SPD, who showed good adherence overall, so this study provides high-quality data allowing for a detailed analysis of the phenomenology of SPD.

Our experience with the assessment of skin picking using EMA and the resulting data serve as a firm basis for further EMA studies on SPD and other disorders in the field of BFRBs and contribute to an enhanced understanding of an understudied but highly impairing mental disorder.

## Abbreviations

BFRB: body-focused repetitive behavior
DSM-5: Diagnostic and Statistical Manual of Mental Disorders, Fifth Edition
EMA: ecological momentary assessment
GAD-7: Generalized Anxiety Disorders-7
*M*_a_: mean automatic style (MIDAS)
*M*_f_: mean focused style (MIDAS)
MIDAS: Milwaukee Inventory for the Dimensions of Adult Skin Picking
PHQ-9: Patient Health Questionnaire-9
SPD: skin picking disorder
SPIS: Skin Picking Impact Scale
SPS-R: Skin Picking Scale-Revised

## Data Availability

The data sets analyzed during this study are not publicly available to protect participant privacy.
